# Hyaluronic Acid-Based Nanocarriers for Anticancer Drug Delivery

**DOI:** 10.3390/polym15102317

**Published:** 2023-05-16

**Authors:** Chao-Ping Fu, Xing-Yu Cai, Si-Lin Chen, Hong-Wei Yu, Ying Fang, Xiao-Chen Feng, Li-Ming Zhang, Chang-Yong Li

**Affiliations:** 1 Institute of Biomaterials and Tissue Engineering & Fujian Provincial Key Laboratory of Biochemical Technology, Huaqiao University, Xiamen 361021, China; 2 College of Materials Science & Engineering, Huaqiao University, Xiamen 361021, China; 3 State Key Laboratory of Molecular Engineering of Polymers (Fudan University), Shanghai 200438, China; 4 School of Materials Science and Engineering, Sun Yat-sen University, Guangzhou 510275, China

**Keywords:** hyaluronic acid, anticancer drug carriers, prodrugs, drug delivery

## Abstract

Hyaluronic acid (HA), a main component of the extracellular matrix, is widely utilized to deliver anticancer drugs due to its biocompatibility, biodegradability, non-toxicity, non-immunogenicity and numerous modification sites, such as carboxyl and hydroxyl groups. Moreover, HA serves as a natural ligand for tumor-targeted drug delivery systems, as it contains the endocytic HA receptor, CD44, which is overexpressed in many cancer cells. Therefore, HA-based nanocarriers have been developed to improve drug delivery efficiency and distinguish between healthy and cancerous tissues, resulting in reduced residual toxicity and off-target accumulation. This article comprehensively reviews the fabrication of anticancer drug nanocarriers based on HA in the context of prodrugs, organic carrier materials (micelles, liposomes, nanoparticles, microbubbles and hydrogels) and inorganic composite nanocarriers (gold nanoparticles, quantum dots, carbon nanotubes and silicon dioxide). Additionally, the progress achieved in the design and optimization of these nanocarriers and their effects on cancer therapy are discussed. Finally, the review provides a summary of the perspectives, the lessons learned so far and the outlook towards further developments in this field.

## 1. Introduction

**Figure 1:**
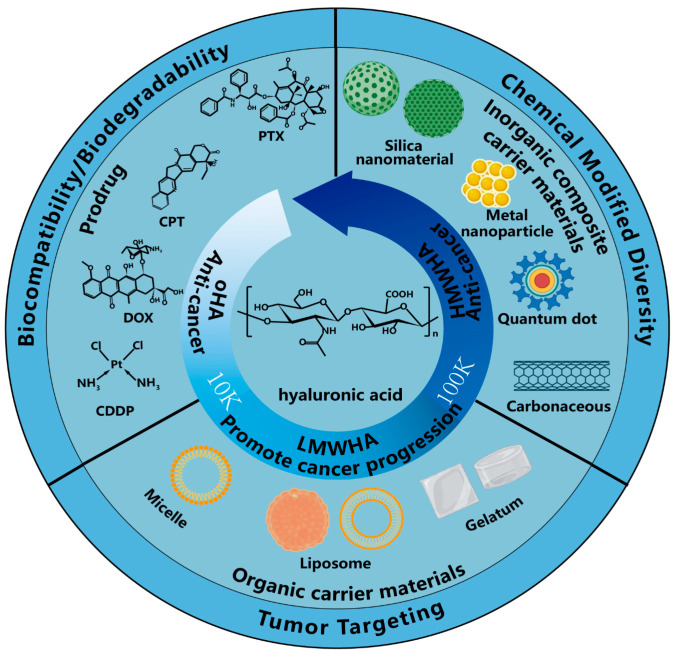
Chemical structure of HA and the hypothesized procancer (promoting cancer growth) and anticancer (preventing cancer growth) activity of HA with different molecular weights and its application.

Hyaluronic acid (HA) is a linear macromolecular mucopolysaccharide composed of D-glucuronic acid and N-acetyl-D-glucosamine connected by β-1,3 and β-1,4 glycosidic bonds (see Figure 1). With its unique molecular structure and physicochemical properties, HA maintains the structure of the extracellular matrix, regulates intracellular activities and participates in the activation and transmission of cell signaling pathways that are associated with inflammation initiation [1], wound healing [2] as well as tumor development and metastasis [3]. The biological function of HA varies with its molecular weight, leading to its division into three categories: HA oligosaccharides (oHAs, <25 disaccharide units), low-molecular-weight HA (LMWHA, 10–100 kDa) and high-molecular-weight HA (HMWHA, >100 kDa). The role of HA in tumor biology is rather complicated; for instance, oHAs and HMWHA were found to inhibit tumor development and metastasis, whereas LMWHA accelerated these processes [4].

The controlled drug release system has been studied extensively since the 1960s to improve the safety, effectiveness and utilization of drugs, and thus reduce the frequency of administration. As an important class of controlled-release preparations, drug nanocarriers were utilized to change the way that drugs enter the body and their in vivo distributions, control the speed of drug release, and deliver drugs to targeted organs. For instance, Lei et al. [5] found that HA can be used as a ligand for surface modification of albumin nanocarriers, which may be used as a drug delivery system in tumor, joint, vitreous and skin tissues. Li et al. [6] found that HA encapsulated in gold nanoparticles could serve as an active targeting drug delivery nanocarrier for the treatment of breast cancer. Ji et al. [7] used HA to modify the surface of curcumin nanocrystals (Cur-NC) to obtain HA@Cur-NCs, which significantly increased the efficiency of Cur utilization, prolonged the retention time of Cur in vivo, and showed better anticancer effects in mice compared to free Cur and Cur-NC. Various types of drug nanocarriers have been developed, such as macromolecular prodrugs, inorganic composite nanomaterial carriers and organic carrier materials. With modification sites, such as carboxyl and hydroxyl groups, HA can easily link to drugs or drug nanocarriers coupled by modifying molecules. HA-based drug nanocarriers are advanced because HA functions in the following aspects: (1) improving the biocompatibility and biodegradability of the drug delivery system, thereby enhancing efficacy and reducing toxicity; (2) enhancing the dispersity of nanocarriers in aqueous solution and improving the drug delivery; (3) constructing tumor-targeted drug delivery systems, as HA is a natural ligand to the endocytic HA receptor, CD44, which is overexpressed in many cancer cells.

HA-based drug nanocarriers have been used in anticancer therapies. Agrawal et al. [8] prepared LPT-HA-NCs by encapsulating nanocrystals of lapatinib (LPT-NCs) with HA to improve their therapeutic efficacy towards triple-negative breast cancer. The results showed that the encapsulated drug carrier exhibited superior anticancer activity to the free drug, and effectively inhibited the metastasis of cancer cells to other sites. Therefore, we have summarized the recent advancements regarding HA-based anticancer nanocarrier families, which include macromolecular prodrugs, inorganic composite nanomaterial carriers and organic carrier materials.

## 2. HA-Based Prodrugs

The concept of macromolecular prodrugs was first introduced by Ringsdorf in 1975 [9] and has since been extensively studied and developed. Common macromolecular prodrugs are composed of water-soluble polymers, small-molecule drugs and modified molecules for the coupling of two such drugs. These drug carriers are effective due to the following factors: (1) they improve the solubility of sparingly soluble or insoluble drugs in aqueous solutions, thus enhancing their bioavailability; (2) they modify the pharmacokinetic behavior of the original drug, extending its half-life in blood and maintaining its activity, hence prolonging the action time of the drug; and (3) they increase the delivery accuracy to target tissues and reduce systemic distributions, thereby reducing the toxicity and side effects.

The first HA-based prodrug, HA Mitomycin (HA-MMC), was prepared by Akima et al. [10] in 1996 by coupling HA with MMC through chemical reactions. HA modification was found to effectively reduce the number of malignant metastatic lung nodules. Thereafter, HA-based prodrugs became increasingly popular in this research area. By linking HA to antitumor drugs using different modified molecules, different HA-based prodrugs can be designed. This section highlights the advances made in the development of HA prodrugs for antitumor therapies, including doxorubicin (DOX), paclitaxel (PTX), camptothecin (CPT) and cisplatin (CDDP).

### 2.1. The Doxorubicin Prodrug

**Figure 2:**
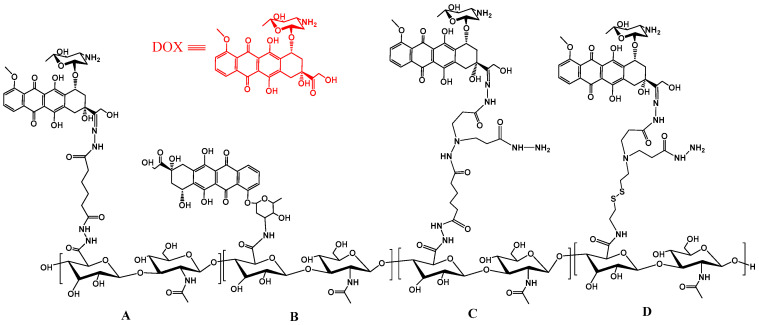
The chemical structure of HA-DOX conjugate. (**A**) DOX is linked to a HA-ADH (adipodihydrazide-modified HA) derivative; (**B**) DOX is directly conjugated to carboxylic groups of HA; (**C**) DOX is linked to a modified HA with dihydrazone groups; (**D**) DOX is linked to a modified HA with disulfide linkage.

Doxorubicin (DOX) is one of the most effective chemotherapy drugs but has high toxicity to the kidneys, liver and heart. HA has been chemically coupled to DOX with different coupling reagents, as shown in Figure 2. By coupling HA (35 kDa) to DOX through the pH-sensitive acylhydrazone bond (Figure 2A), Cai et al. [11] successfully synthesized an acid-sensitive hyaluronan-doxorubicin (HA-DOX) prodrug with different drug loadings (5–15%, *w*/*w*). Histological analysis showed that the prodrug not only effectively reduced the cardiotoxicity and side effects of DOX but also delayed tumor progression (by about 10 weeks) and improved the survival rate of laboratory animals. Moreover, Oommen et al. [12] prepared another HA-DOX prodrug via amide linkage of the 3′-amino group of DOX and the carboxyl group of HA (150 kDa) using carbodiimide coupling chemistry (Figure 2B). In vitro cell experiments showed that this prodrug was far less toxic to mouse embryonic fibroblast NIH3T3 cells and human breast cancer MCF-7 cells (CD44 low-expression cell line) than DOX but was highly toxic to human colon cancer HCT116 cells (CD44 high-expression cell line). In addition, an HA-DOX prodrug responding to both pH and reducing environments was synthesized by conjugating DOX to an HA backbone through disulfide linkages and hydrazone bonds (Figure 2C,D) [13]. Through the activation of tumor-microenvironment-triggered drug release, the dual-stimuli-response HA-adriamycin prodrug exhibited the strongest cytotoxicity and apoptosis-inducing ability among all the tested groups. A multifunctional biopolymer–anticancer drug combination nanomedicine has been designed by Zhang et al. [14], consisting of oHAs and DOX co-loaded in a polymer-lipid hybrid nanoparticle functionalized with an internalizing cyclic peptide iRGD, which shows promise in inhibiting primary triple-negative breast cancer tumors and preventing spontaneous metastasis to the lungs and lymph nodes in a mouse model.

### 2.2. The Paclitaxel Prodrug

As a broad-spectrum anticancer drug, paclitaxel (PTX) can be used to treat a variety of cancers, including ovarian, breast, lung, prostate, esophageal and cervical cancers, as well as melanomas, Kaposi’s sarcoma and other types of solid tumors. However, clinical applications of PTX are limited due to its strong hydrophobicity and adverse side effects [15].

**Figure 3:**
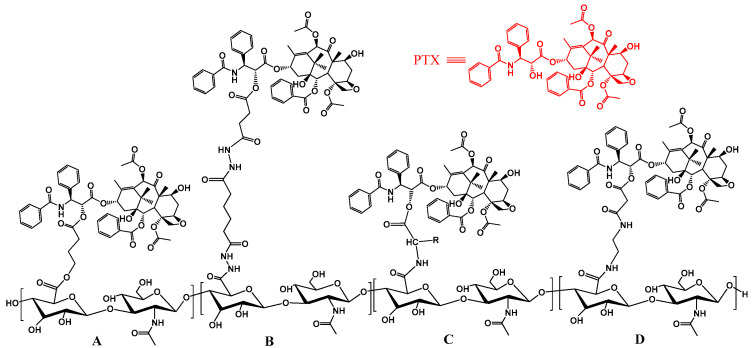
The chemical structure of the hyaluronan-PTX conjugate. (**A**) A hemisuccinate N-hydroxysuccinimide (NHS) activated ester of PTX is linked to an HA-ADH derivative; (**B**) A 2′-PTX-4-bromobutyrate is conjugated to HA; (**C**) An amino acid-PTX derivative is linked to HA activated by carbodiimide; (**D**) A hemisuccinate NHS activated ester of PTX is linked to an HA-ethylenediamine derivative.

Rosato et al. [16] prepared an HA PTX prodrug (HYTAD1-p20) by coupling 4-bromobutyric acid with HA (200 kDa) and PTX (Figure 3A). The solubility of PTX in aqueous solutions was increased by nearly 500 times, and the prodrug showed stronger cytotoxicity as well as a higher uptake by cells.

Moreover, the Prestwich research team proposed another route for the preparation of an HA PTX prodrug (HA-Taxol) in 1999, as shown in Figure 3B [17]. The group chemically coupled the 2’-OH of PTX with the HA-ADH. They also prepared a fluorescent (BODIPY)-labeled HA derivative for in vitro cell experiments. Thereafter, a series of HA-Taxol-based functional derivatives were prepared, and their antitumor effects in different tumor mouse models were evaluated (Figure 3C,D) [18]. The models included non-resistant (SKOV3ip1) and resistant (HeyA8-MDR) ovarian cancer models, as well as head and neck squamous cell carcinoma models. Modification of HA enhanced the sensitivity of tumor cells to PTX in the drug-resistant ovarian cancer models, hence alleviating the resistance of HeyA8-MDR cells to the drug.

### 2.3. The Camptothecin Prodrug

Camptothecin (CPT, a Chinese tree derivative) affects topoisomerase I, allows DNA cleavage but inhibits subsequent ligation, therefore resulting in DNA strand breaks. However, CPT has poor solubility in water, and the lactone ring in its molecular structure is unstable in aqueous solutions. This makes the drug prone to breakdown, reducing its activity. Therefore, a series of CPT prodrugs have been developed in order to improve the solubility and stability of CPT in aqueous solutions, including gemcitabine [19] and irinotecan [20], which have already been used in cancer chemotherapy.

**Figure 4:**
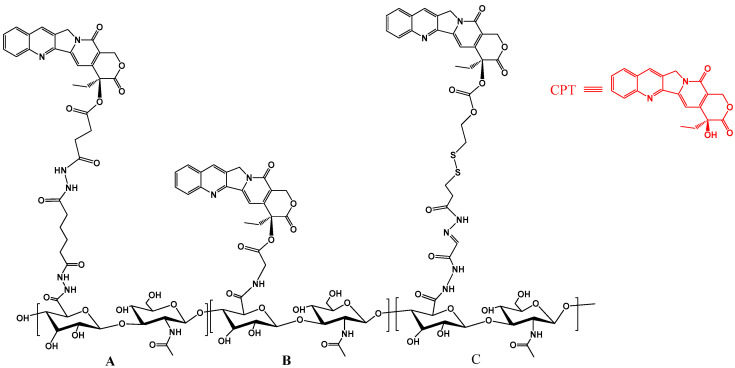
The chemical structure of the hyaluronan-CPT conjugate. (**A**) An amino acid-CPT derivative is linked to an HA-ADH derivative; (**B**) CPT is directly conjugated to carboxylic groups of HA; (**C**) CPT is linked to a modified HA with disulfide linkage.

Xu et al. [21] selected two HAs with different molecular weights (8 kDa and 100 kDa) to modify CPT, as illustrated in Figure 4A. The two hyaluronic CPT prodrugs (HA-CPT-8k and HA-CPT-100k) were prepared by derivatization with adipic acid dihydrazide (ADH). The reactivity of HA increased with the activated CPT-NHS ester N-hydroxysuccinimide. After modifications, the solubility of CPT in aqueous solutions increased from 2.08 µg/mL to 420 µg/mL and 620 µg/mL for HA-CPT-8k and HA-CPT-100k, respectively. Additionally, HA-CPT-100k showed a higher drug loading capacity than HA-CPT-8k. In the study, human liver cancer HepG2 cells which express CD44 and human ovarian cancer A2780 cells were employed to investigate the cellular uptake and the anticancer effect of HA-CPT. The results showed that HepG2 cells had a higher uptake of both HA-CPT-8k and HA-CPT-100k and that the A2780 cell uptake of HA-CPT-100k was much lower than that of HA-CPT-8k. Moreover, the drug clearance analysis showed that the CPT entering the cell through diffusion and penetration was quickly cleared from the cell (4 h). In contrast, the concentration of HA-CPT synthesized by directly modified CPT (with HA) reached its peak after two hours of incubation, but only 20% of the prodrug had been cleared (as shown in Figure 4B). This finding showed that the prodrug effectively prolonged the residence time of CPT in the cells.

In addition, Yang et al. [22] designed a new synthetic route for hyaluronan CPT prodrugs, as depicted in Figure 4C. The novel approach involved substituting 5% of the carboxyl groups in HA with the aldehyde groups. Thereafter, the reactant was reacted with L-Tartaric acid bishydrazide modified CPT and then coupled with a cholesterol group to synthesize a cholesterol group-containing hyaluronanic CPT prodrug molecule capable of self-assembling into nanoparticles in the solution. The resulting nanosized CPT prodrug accumulated in tumor tissues in vivo, demonstrating enhanced tumor-targeted drug delivery capabilities compared to general macromolecular prodrugs, due to its high permeability and retention effect (EPR effect) in solid tumors.

### 2.4. Cisplatin

The prodrug cisplatin (cis-dichlorodiamminoplatinum (II) or CDDP), a platinum-containing anticancer drug that belongs to the class of cell cycle non-specific drugs, is commonly used to treat cancer. Although the drug has significant antitumor efficacy, high doses are toxic to the kidneys and liver and are associated with the inhibition of bone marrow functions [23,24]. Forrest and his research team [25] attempted to reduce the toxicity and side effects of CDDP and improve its drug utilization rate by designing and synthesizing an HA-based CDDP prodrug (HA-CDDP), then studied the behavior of the released drug. Their results demonstrated that HA-CDDP significantly increased the drug’s cumulative concentration in tumor tissues, which led to sustained release behavior. Additionally, the drug had an action time of up to 96 h within the effective concentration range; meanwhile, the associated damage to the liver, spleen and kidney was significantly reduced. Notably, in addition to the above antitumor drugs, HA has also been used to modify some compounds with antitumor activities, including butyric acid [26] and quercetin [27]. This subsequently improves the solubility of these compounds in water and reduces systemic toxicities, thereby increasing their antitumor activity.

## 3. HA-Based Inorganic Composite Carrier Materials

HA is widely applied in the modification of inorganic nanomaterials, including quantum dots (usually cadmium sulfide and cadmium selenide); metal nanoparticles, such as gold nanoparticles; metal oxide nanoparticles, such as iron oxide nanoparticles; carbonaceous materials, such as fullerene and carbon nanotubes; and siliceous materials, such as mesoporous silica.

### 3.1. Metal Nanoparticles

Gold nanoparticles (AuNPs) have unique optical properties and chemical inertness, making them easily modifiable on their surfaces, and are thus increasingly popular in various fields, including drug and gene delivery, immunotherapy, photothermal therapy, biosensors and photoacoustic imaging. They are also being gradually introduced into other fields, such as intelligent diagnosis and treatment [28]. Cao et al. [29] reported that an HA-modified AuNP cancer vaccine could effectively evoke antitumor immune responses in mice and inhibit tumor growth. Specifically, the authors simultaneously modified gold nanoparticles with a sulfhydrylated HA (HA-SH) and a sulfhydryl-containing antigen (ovalbumin, OVA), resulting in a stable dispersion and good water solubility of the gold nanoparticle solution (HA-OVA-AuNPs). The nanoparticles could specifically be recognized and taken up by dendritic cells, thereby upregulating CD44 receptors, generating active oxygen molecules under near-infrared laser-mediated irradiation and producing a local thermal effect. Consequently, this action accelerated destruction of the lysozyme and enhanced the activity of protein groups as well as the presentation of downstream MHC I antigens, ultimately leading to activation and response of tumor-specific cancer-killing CD8^+^ T cells. Notably, CD8^+^ T cells are important components of the human immune system and can effectively remove virus-infected and cancer cells.

Another type of metal nanoparticle, iron oxide nanoparticles or superparamagnetic iron oxide nanoparticles (SPIONs), commonly used in magnetic resonance imaging, have also been extensively studied. Li et al. [30] developed polyethylenimine (PEI) iron oxide nanoparticles by modifying them with two different molecular weights of HA (6 and 31 kDa), and then tracked in vivo distributions after intravenous injections into the tails of mice. The results indicated that although the liver and spleen were significantly affected, the nanoparticles modified with the 31 kDa HA exhibited superior antitumor effects compared to the 6 kDa counterparts. On the other hand, Zheng et al. [31] developed a multifunctional micellar dual tumor-targeted drug delivery platform based on HA micelles co-encapsulated with the therapeutic agent docetaxel and iron oxide nanoparticles. This multifunctional micelle is capable of converting light into heat under near-infrared light irradiation conditions to further achieve thermal therapy temperatures and induce photothermal ablation of breast cancer cells. This finding is expected to improve the efficiency of combined photothermal chemotherapy.

### 3.2. Quantum Dots

Quantum dots (QDs) are nanomaterials with unique luminescent properties. They mainly include cadmium sulfide, cadmium selenide and cadmium telluride, and have an emission wavelength that generally ranges from 650 to 800 nm. When used in vivo, it is necessary to modify ligands or organic compounds on the surface of QDs for safer and more stable molecular imaging. For instance, Kim et al. [32] chemically modified the surfaces of QDs, then coupled them with different amounts of HA to obtain HA-modified QDs (HA-QDots). Animal experiments revealed that a small amount of HA-modified QDs were distributed throughout the body, while sufficient levels were observed across various tissues, with the liver having the highest concentration. Overall, they concluded that this might be related to the presence of a large number of HA receptors in the liver. Similarly, Hou et al. [33] prepared HA-modified porous silica (pSiO_2_) nanocarriers based on a two-phase method, a carrier with large channels and high loading capacity (29.3%). Ag_2_S quantum dots were then embedded into the porous structure of the pSiO_2_ carriers to impart good photothermal effects to the carriers. This facilitates the carriers’ reactive drug release and combined photothermal chemotherapy.

### 3.3. Carbonaceous Nanomaterials

Carbonaceous nanomaterials have attracted extensive research interest, among all inorganic nanomaterials, owing to their exceptional mechanical, thermal and optical properties. Notably, some carbonaceous nanomaterials, such as carbon nanotubes, have been shown to produce fluorescent or acoustic signals used for imaging, in a similar fashion to AuNPs, and also have photothermal conversion effects which are useful for photothermal therapy in the visible and infrared regions [34]. However, their clinical applications are constrained by the biocompatibility of carbonaceous materials.

**Figure 5:**
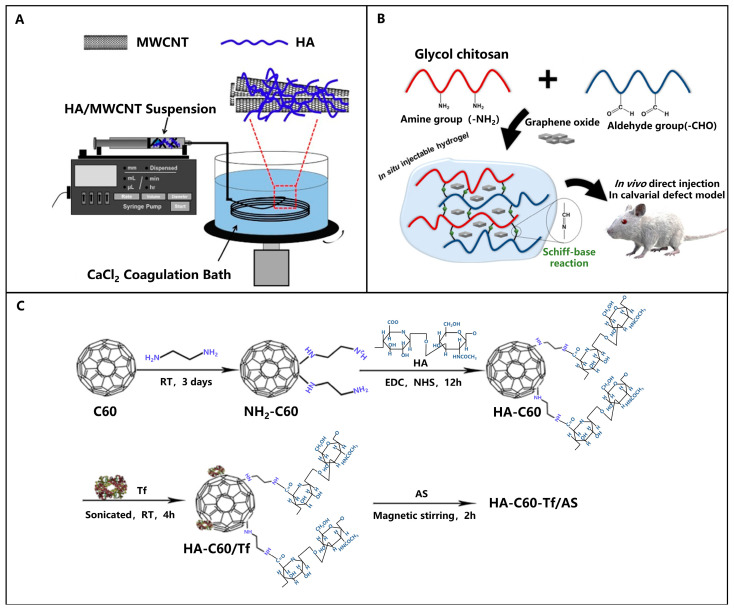
Schematic diagram of the mechanism based on HA-modified carbonaceous nanomaterials. (**A**) Hybrid microfibers composed of HA and multi-walled carbon nanotubes (MWCNTs) were prepared using wet spinning - reprinted from [36]. (**B**) Schematic representation of hydrogel preparation and in vivo action - reprinted from [37] with permission from Elsevier (2020). (**C**) Schematic diagram of the preparation process of HA-C60-Tf/AS - reprinted from [38] with permission from Elsevier (2015).

Carbonaceous nanomaterials can also be subdivided into fullerene (C60), nanotubes, nanodots, nanodiamonds and graphene derivatives, based on their sizes and shapes. Datir et al. [35] developed an HA-modified multi-walled carbon nanotube and used the hydrophobic cavity of the nanotube to load anticancer drugs, such as DOX. Zhang et al. [36] investigated the effects of different variables on the fabrication of hybrid microfibers composed of HA and multiwalled carbon nanotubes, resulting in high-performance microfibers with potential applications in various fields. Lee et al. [37] constructed an injectable hydrogel system based on graphene oxide using glycol chitosan and oxidized hyaluronic acid, which is expected to have applications in bone tissue defects (Figure 5B). Zhang et al. [38] prepared a multifunctional tumor-targeting drug delivery system, HA-C60-Tf, with good water solubility and tumor-targeting activity. This delivery system can greatly enhance the pharmacological activity of the drug at the target site.

### 3.4. Silica Nanomaterials

Mesoporous silica nanoparticles (MSNPs) are gradually gaining popularity in the fabrication of biofunctional materials owing to several advantages, such as adjustable pore size, large capacity, excellent chemical stability, good biocompatibility and ease of synthesis [39]. Ricci et al. [40] recently coupled two forms of HA with different molecular weights of 6.4 kDa and 200 kDa to aminopropyl functionalized MSNPs, then compared the physical, chemical and biological properties across the modified MSNPs. Their results showed that HA-based modification significantly improved the stability of silica dispersion, as evidenced by higher cellular uptake in MSNPs modified with HMWHA. Prior to this, Yu et al. [41] had used HA-MSNPs loaded with the antitumor drug DOX to construct a targeted drug delivery system. The researchers had further evaluated the associated cytotoxicity in vitro, the uptake capacity by human colon cancer HCT-116 cells, and then explored HA-MSNPs’ potential value in drug delivery.

## 4. HA-Based Organic Carrier Materials

### 4.1. Micelles

Micelles have recently become the focus of several studies, especially for the delivery of antitumor drugs prepared by amphiphilic polymer micelles. Functionally, amphiphilic polymers are capable of self-assembling into nanomicelles with a core–shell structure and hence have excellent in vitro stability in aqueous solutions. In addition, amphiphilic polymer micelles can be enriched in tumor tissues in vivo through the EPR effect (passive targeting); thus, they can be used to target tumors owing to their ability to bind a specific ligand. Moreover, the hydrophobic inner core of the polymer micelle can carry most hydrophobic antitumor drugs, while the hydrophilic shell is usually helpful in avoiding removal by the reticuloendothelial system (RES). On the other hand, water-soluble drugs can be loaded by electrostatic adsorption or chemical modification onto the hydrophilic shell of the micelles, thereby allowing the co-loading of multiple drugs. Although micelles have been found to improve the selectivity of drugs to tumor cells, they also have a number of limitations, such as low drug loading rate, poor encapsulation efficiency and in vivo instability. Specifically, they can be easily diluted by blood if their concentration is below the critical concentration of micelles and may also react with blood components. Therefore, selecting appropriate polymers is imperative for the accurate preparation of multifunctional nanomicelles with fewer limitations.

Amphiphilic polymer micelles made with HA as a raw material can not only reduce the phagocytosis of polymer micelles by RES but also achieve active targeting of tumor cells and overexpression of CD44 (Table 1). Additionally, the molecular structure of HA contains numerous reactive groups, such as carboxyl and hydroxyl groups, which are conducive to the construction of multi-functional drug delivery systems. Moreover, a block polymer can be formed through reductive amine reaction by coupling with a primary amine-containing compound on the terminal group of HA [42]. Yao et al. [43] used a reductive amination reaction to attach the amine group of distearoylphosphatidylethanolamine (DSPE) to the aldehyde group of the reducing end HA, producing a DSPE-HA single-site coupling. This coupling was then used as an intrinsic ligand for the CD44 receptor, and the products were referred to as targeted glioma-associated oncogene homolog 1 (Gli1) siRNA nanoparticles. Tests using an in vitro tumor model simulated by gastric cancer stem cells (CSCs) showed that targeting Gli1 siRNA nanoparticles significantly reduced the expression of Gli1 protein and effectively inhibited the formation of CSC tumor spheroids.

### 4.2. Liposomes, Transfersomes, Niosomes and Ethosomes

**Figure 6:**
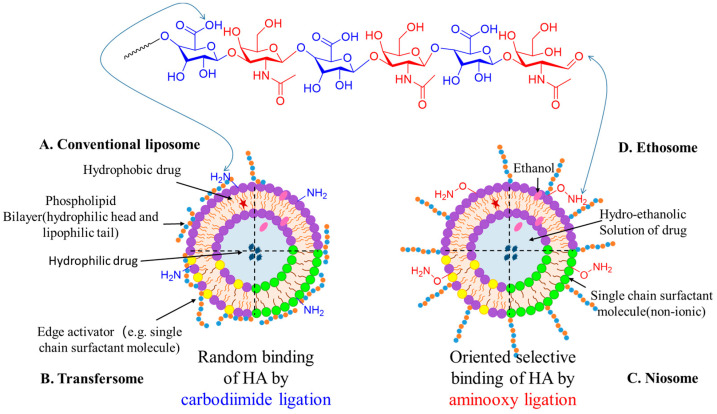
Schematic representation of random and oriented selective binding of HA to (**A**) liposomes, (**B**) transfersomes, (**C**) niosomes and (**D**) ethosomes. Picture adapted from [64].

Different kinds of vesicular formulations have been investigated as drug carriers: liposomes, transfersomes, niosomes and ethosomes. To date, some liposome drugs, such as Myocet^®^ and Doxil^®^, have been approved by the FDA for marketing [60]. As drug delivery systems, liposomes offer several advantages: (1) their lipid molecular layers and hydrophilic inner cores can allow the loading of hydrophobic and hydrophilic drugs, respectively; (2) they have excellent biocompatibility and biodegradability; and (3) they have low toxicity and immunogenicity. Previous studies have reported that cationic lipids can be combined with DNA via electrostatic action for gene therapy. Cationic nanocarriers can induce cell necrosis, which will limit their use in clinical applications to some extent. Qian et al. [61] prepared cationic liposomes (HALPs) modified with a mass fraction of 10% HA. Compared to unmodified liposomes, cationic liposomes modified with HA showed low cytotoxicity due to the blocked surface charge and significantly reduced lung inflammation when applied to mouse lungs. This vector is expected to be a less toxic and more efficient gene carrier for tumor targeting. In a study by Amirreza et al. [62], a material was developed by combining epirubicin (Epi) and nylon nanoparticles (Nios) modified with HA. This Epi-Nio-HA nanocomplex was found to significantly reduce the volume of mammary tumors in mice by 28% compared to Epi alone, without any adverse effects on the liver and kidney. The functionalization of niosomes with HA modification provides a promising nanoplatform for the targeted delivery of epirubicin. Recently, Bartheldyova et al. [63] demonstrated the coupling of HA with liposomes via different functional groups, and Figure 6 presents a schematic representation of the stochastic or directional selective binding of HA to liposomes, transfersomes, niosomes and ethosomes.

### 4.3. Gelatin

The unique chemical properties of HA have recently led to its being studied as a component of gel systems, owing to its lack of immunogenicity, biocompatibility, biodegradability and unique water-retaining properties. Consequently, HA-based hydrogels have attracted considerable attention in the fields of cell therapy, regenerative medicine, wound healing, molecular delivery, tissue engineering as well as tumor diagnosis and treatment. Common HA gels have complex 3D cross-linked network structures, which enhance the persistent release of drug-based or other active-molecule-loaded drugs through physical embedding [65] or chemical coupling [66]. Burdick and Prestwich [67] have summarized research advances and applications of different forms of HA gels in biomedicine. Notably, nanogels and injectable hydrogels have unique advantages in drug delivery to tumor tissues.

Moreover, Xu et al. [68] developed an injectable hydrogel comprising interferon α2a (IFN-α2a) loaded with HA (90 kDa) tyramine (HA-Tyr). They found that the concentration of IFN-α2a released by the HA-Tyr hydrogel was three times higher in plasma and tumor tissues after subcutaneous injection than with the administration of IFN-α2a solution alone, and this effectively inhibited tumor growth as well as angiogenesis in tumor tissues. HA gels can also be loaded with DNA and siRNAs and applied in antitumor therapy. For instance, Segura et al. [69] prepared an HA fibrin composite hydrogel, then stably and uniformly dispersed the DNA carrier complex in the hydrogel scaffold to avoid aggregation and inactivation in vitro. They found that the gene vectors released by the hydrogel had high transfection efficiency both in vivo and in vitro, and hence could be applied for the delivery of non-viral gene vectors.

### 4.4. Other Organic Carrier Materials

HA has also been used in the modification of microbubbles [70,71], electrospun membranes [72], cationic polymers [73] and medical adhesives [74]. For instance, Cerroni et al. [70] used HA (700,000 g/mol) to modify polyvinyl alcohol (PVA) microbubbles and found that the resulting air-filled cores of the PVA microbubbles could be imaged under ultrasound. In addition, HA-based modification reduced the microbubbles’ cytotoxicity and increased their uptake by tumor cells. The generated microbubbles could effectively be loaded with antitumor drugs and have potential in the construction of an integrated tumor diagnostic and treatment platform. Previous studies have also shown that HA modified cationic polymers can target tumors, shield part of the positive charge, improve transfection efficiency and increase the stability of the cationic-polymer gene carrier, while reducing its toxicity. For example, Zhang et al. [73] used PEGylated HA to modify a gene carrier constructed using a cationic polymer and polycaprolactone (PCL) and found that hyaluronidase could effectively degrade HA as well as reverse the charge of the complex. This subsequently allowed DNA to escape from the lysosome and improve the efficiency of the DNA and gene transfection. Moreover, HA can be used to modify biological macromolecules, such as proteins, peptides and nucleotides.

## 5. Conclusions and Outlook

Numerous research efforts have been dedicated to exploring the potential of HA and its derivatives as antitumor drug carriers for targeted delivery. HA has excellent tumor-targeting ability, good biocompatibility and biodegradability, and can be chemically modified in diverse ways. At present, most studies are still in the stage of in vitro experiments, and only a few have entered the stage of clinical trials. The current advancements in molecular biotechnology and nanotechnology are expected to significantly popularize HA and its derivatives as excellent drug carriers and enhance their application in tumor diagnosis and therapy. In contrast, oHAs with MWs below ~10 kDa have been found to reduce the adhesion, migration, invasion and proliferation of cancer cells, possibly by antagonizing oHA receptors. oHAs can sensitize cancer cells to chemotherapeutic drugs, including DOX, suggesting their great potential for systemic combination therapy. Notably, most research to date has focused on HMWHA, whose function is relatively simple. Therefore, further explorations are required to unravel the underlying mechanisms of in vivo actions associated with HAs of different molecular weights, provide a clear understanding of HA’s biological activity and exploit its activity for the delivery of antitumor drugs. Furthermore, efforts should be directed towards the precise design of intelligent drug carriers and integrated carriers for effective cancer diagnosis and treatment.

While research on HA-based nanomedicine has rapidly developed, the translation of preclinical studies into clinical efficacy has been limited. The observed differences in efficacy may be attributed to species differences and limitations in animal disease models that fail to fully recapitulate human malignancies, particularly in the context of cancer research, where differences in tumor size, growth rate, and microenvironment must be considered. Therefore, careful reevaluation of drug release and dosing regimens in animal models is necessary to ensure successful translation to human patients. Future advancements in the field will require a better understanding of the underlying mechanisms of HA’s biological activity and the design of intelligent drug carriers and integrated carriers for effective diagnosis and treatment of cancer. There is also a need to explore the potential of HAs of different molecular weights and their in vivo actions, which may lead to new therapeutic approaches. Knowing the different courses of action before, during and after the application of HA-based nanomedicine is crucial for ensuring the safety and efficacy of these therapies. This includes understanding the pharmacokinetics and pharmacodynamics of these agents, as well as monitoring for potential adverse effects.

## Figures and Tables

**Table 1:** Summary of HA derivatives used in the preparation of micelles.

Component	Loaded Drug	Average Particle Size (nm)	Cell Type	Ref.
HA-NB-SC	DOX	139	HeLa	[44]
Galactosamine-HA-Vitamin E succinate	NCTD	199.2	MCF-7, HepG2, MCF-7/Ard	[45]
Acetylated-HA-perfluorocarbon-Pyropheophorbide a	N/A	155	OM431	[46]
HA-poly(lactide)-sectorial poly(amidoamine)-docetaxel	Docetaxel	N/A	MCF-7	[47]
mPEG-HA-(deoxycholic acid)-N-acetyl-L-cysteine	PTX	147	MCF-7, H22	[48]
HA-ss-ibuprofen	DOX	120	MCF-7, 4T1, NIH 3T3	[49]
HA-ss-curcumin	C6	74.2	B. End3, G422	[50]
HA-ss-mercaptopurine	6- Mercaptopurine	264.4	B16F10	[51]
PTX/folic acid-hyaluronic acid-SS-vitamin E succinate	PTX	148.8	MCF-7, NIH3T3	[52]
HA-dopamine-Cu-mercaptopurine	6- Mercaptopurine	173.5	A549, NIH3T3	[53]
Folic acid-HA-α-tocopherol succinate	PTX	135	MCF-7, H22	[54]
HA-g-polyethylene glycol methyl ether-polymers	DOX	116.65	MCF-7, CT26	[55]
HA-cystamine dihydrochloride-stearic acid	DOX	N/A	HCT116, CT26, HEK293	[56]
HA-deoxycholic acid-histidine -Pluronic F127	DOX	218.7 (carrier/DOX = 5/1)	MCF-7, MCF-7/Adr	[57]
N-Deacetylation of HA, dodecylamine, DOX	DOX	N/A	MCF-7	[58]
HA-b- poly (d,l-lactide-co-glycolide) copolymer	N/A	213.4	A549	[59]

## Data Availability

Not applicable.
